# Development and validation of a questionnaire to test Chinese patients’ knowledge of inflammatory bowel disease

**DOI:** 10.1038/s41598-023-34286-6

**Published:** 2023-04-30

**Authors:** Huabing Xie, Jixiang Zhang, Chuan Liu, Bingxiang Yang, Weiguo Dong

**Affiliations:** 1 Department of General Practice, Renmin Hospital of Wuhan University, Wuhan, 430060 China; 2 Department of Gastroenterology, Renmin Hospital of Wuhan University, Jiefang Road 238, Wuhan, 430060 China; 3 Department of School of Nursing, Wuhan University, Wuhan, 430062 China

**Keywords:** Inflammatory bowel disease, Intestinal diseases, Patient education

## Abstract

A good understanding of a disease facilitates patient-centered management. We aimed to develop and validate a questionnaire to assess inflammatory bowel disease (IBD)-related knowledge and analyze the factors affecting patients’ knowledge of IBD. We invited 15 experts to develop and modify an IBD knowledge questionnaires and 709 patients to test the reliability and validity of the questionnaires as well as analyze the factors related to the disease knowledge of patients with IBD. In internal consistency, Cronbach’s α coefficients for the common items, ulcerative colitis (UC), and Crohn’s disease (CD) knowledge questionnaires were 0.886, 0.89, and 0.886, respectively. In cross-item consistency, Spearman-Brown split coefficients of the common items, UC, and CD knowledge questionnaires were 0.843, 0.812, and 0.812, respectively. In time consistency, the test–retest reliability ICC was 0.862 (*P* < 0.001). The correlation between researcher scores, IBD-KNOW scores, and the original questionnaire scores was greater than 0.7 (*P* < 0.001). Multiple linear regression demonstrated that the factors, including disease type, age, body mass index, education level, income, treatment cost, duration of disease, and frequency of visits, affected the IBD patients’ knowledge of the disease (*P* < 0.05). The IBD knowledge questionnaires had good reliability and validity and, therefore, can be used to assess patient knowledge of the disease.

## Introduction

Inflammatory bowel disease (IBD) is a group of chronic gastrointestinal inflammatory diseases, including ulcerative colitis (UC) and Crohn’s disease (CD). IBD was formerly considered a Western disease. However, the increasing prevalence of IBD since the start of the twenty-first century in recently industrialized countries^1,2^ has made it a global public health challenge^3^. There is currently no cure for IBD, and it affects almost every aspect of life, including the private, professional, and social lives of the patients^4^. The prevalence, incidence, disability-adjusted life years, and years of life with disability due to IBD in China have increased in recent years^5^. Thus, ways to better treat and manage IBD have become a big issue, especially given the chronic, early onset, and relatively low mortality rate of patients with IBD^2^.

With the transformation of medical models, the patient-centered chronic disease management model has received increased recognition. One important and emerging model is population health management (PHM)^6^, and self-management is an important part of PHM^7^. Only when patients understand IBD correctly can they achieve good self-management and transition from sudden, accidental, and passive diagnoses and therapy to active, planned, and individualized long-term care^8^. The treatment and management of IBD are complex. New treatments and treatment strategies continue to emerge, making it difficult for even the most experienced providers to stay current and provide the best care. Structured health education may be an effective way to address this issue. Previous studies demonstrated the benefits of enhancing patient knowledge, including an improved ability to cope with the disease^9^, adherence to medication^10,11^, and improved disease outcomes^12^, as well as decreased healthcare costs^3,13^.

The effectiveness of patient health education needs to be assessed by a proven tool.

The questionnaires currently available to assess IBD include The Crohn’s and Colitis Knowledge^14^ (CCKNOW), IBD-KNOW^15^, IBD-INFO^16^, and U-IBDQ^17^ questionnaries, all of which have specific advantages but also different problems. For example, the IBD treatment part of the CCKNOW questionnaire has not been updated, the IBD-Know questionnaire lacks a knowledge assessment of IBD detection means, and rarely includes diet and nutrition content, the length of the IBD-INFO questionnaire may reduce the willingness of patients to participate, the physiological assessment of U-IBDQ disease is too complex for patients, and the social rights part of the response of patients is not suitable for cross-cultural applications. Given the above reasons, we wanted to develop a short IBD knowledge questionnaire with different versions for CD and UC, including basic information as well as information on diet and nutrition, treatment, disease surveillance, and disease evaluation, and investigate the factors that influence IBD patient knowledge to objectively assess patient concerns.

## Methods

### Development of the questionnaire

First, a five-member research group, including two IBD specialists, one nursing expert, and two doctoral students who were familiar with IBD-related knowledge, was established to design the original questionnaires. The items on the questionnaires were determined according to the principles of flexibility, practicality, decomposition, and traceability. By referring to relevant literature, the content contained four dimensions, A: pathology and risk factors; B: diet and nutrition; C: therapy; and D: disease surveillance and special circumstances. Both single-choice and multiple-choice questions were included. Every question was with multiple choices and included an “I don’t know” option. Correct answers to multiple choice questions were worth 2 points, partially correct answers were worth 1 point, and wrong answers were worth 0 points. One point was given for a correct answer to a single-choice question, and 0 points for a wrong answer.

The original questionnaires were evaluated and modified using Delphi method^18^ by the specialists. The specialists’ criteria were: ① clinical medical experts, psychological experts, and nursing experts in the IBD field, ② more than five years of clinical experience in the IBD field, and ③ an intermediate technical title or above. Studies have shown that good reliability can be achieved when the number of advisers is about 15^19^. The expert consultation questionnaire contains three parts: ① preface, including the research purpose, research methods, and notes for completing the questionnaire; ② questions on the basic information of specialists, including the general information of experts and the degree of familiarity with the survey content, and ③ an item inquiry form, including the importance, relevance, clarity score, and comment column for each dimension item. The Likert 5-level rating method was used for scoring. The specialists were consulted in April 2022. The inclusion criteria for the items^18^ were a mean importance score and correlation score greater than 4.0 and a coefficient of variation (CV) less than 0.25. The criteria for modifying entries were: ① the meaning of entries was unclear or not exact, ② entries were complicated to comprehend, and ③ the specialists believed there were other good reasons to modify entries. The criteria for adding entries were: ① entries could supplement insufficient parts of the theoretical, conceptual framework or deficiencies in the existing item pool measurement dimension or degree (depth or width), and ② the specialists believed that there are other good reasons to add entries.

### Questionnaire revision and reliability and validity test

Forty-one subjects were recruited from May to June 2022 in the Department of Gastroenterology, Affiliated Hospital of Wuhan University. All of them were older than 18, with normal thinking and sufficient energy. After obtaining informed consent, they were invited to attend the test revision of the first version of the questionnaires. The study doctor interviewed the subjects for about 15 min after they completed the preliminary questionnaires, the Chinese IBD-KNOW^9^, and provided general demographic information. The interview outline was based on the design of the IBD knowledge questionnaires we developed. The research physician evaluated the patients’ understanding of IBD by asking standardized questions about the dimensions measured by the questionnaires. The same research physician assessed each dimension, and the final score was the sum of the scores of each dimension (range 0–40). At the end of the assessment, the participants were asked whether the items in the questionnaires had covered the IBD topics they were interested in and whether the items were clearly expressed and easy to grasp. They were encouraged to make suggestions for modifications. They were asked to give a self-score (0 – 10 points) of their knowledge of IBD. The questionnaires were modified according to patient feedback, excluding items to which 95% of patients responded correctly in the first version to avoid the ceiling effect (n = 39), and the correlation between the scores of each measurement tool was computed.

### Formal research

The final version of the IBD knowledge questionnaires was delivered to patient groups From July to August 2022 at several IBD centers across the country to recruit participants through the Questionnaire Star platform (a professional online questionnaire survey, examination, evaluation, and voting platform). The questionnaires also reported the purpose of the research and the principles of confidentiality and knowledge. In addition to the IBD knowledge questionnaires, other informations were gathered, including age, gender, body mass index (BMI) (underweight (BMI < 18.5 kg/m^2^), normal (BMI 18.5–24 kg/m^2^), overweight (BMI > 24 kg/m^2^)), smoking status, drinking status, exercise habits, marital status, educational level (educational level (low, high school or below; intermediate, college; or high, Bachelor degree or above), work, living status, diagnosis (UC or CD), duration, disease activity, the influence of IBD on work or school (mild, no influence/retired/long-term unemployed; moderate, frequent absences due to bouts of IBD; and severe, unemployed/out of school due to IBD), treatment history, surgery for IBD, IBD-related complications, main sources of IBD information, and treatment compliance. Disease activity was evaluated by the simplified Colitis Clinical Activity Index (SCCAI) for UC patients and the simplified CD activity index (CDAI) for CD patients. Therapy compliance evaluation involved five aspects: “adjusting diet,” “avoiding bad living habits,” “monitoring nutritional status,” “implementing a treatment plan,” and “regularly reviewing,” using the Likert 4-grade evaluation method as “never,” “occasionally,” “normal,” and “always,” in which were assigned 1–4 points, respectively.

After 1 to 2 weeks of the formal investigation, we selected some participants for retesting and evaluation of questionnaires. The assessment content covered four aspects: “proper difficulty of the item,” “clear expression of the item,” “comprehensive item content,” and “beneficial to understanding IBD.” The Likert 5-level scoring method was used to assign 1 to 5 points corresponding to “strongly disagree” to “strongly agree,” respectively. When the mean of the evaluation results for all aspects was greater than 3.5, and the CV was less than 0.25, patients were considered to have a high agreement with the questionnaires.

### Study population

According to the international principles of psychometrics and questionnaire design, the recommended sample size is 5 to 20 times the number of items in the questionnaire. About 10% of the questionnaires issued were withdrawn or invalidated; therefore, the sample size needed to be 5.5 times the number of questionnaire items. As a result, a sample size of more than 302 was needed.

The criteria for the inclusion of patients were as follows: (1) the patients with confirmed IBD; (2) the patients with age ≥ 18 years; (3) the patients, who understood and agreed to be investigated. The patients with no internet access, cognitive impairment, or those, who did not provide informed consent, were excluded.

### Ethical statement

This survey was conducted according to the principles of the Declaration of Helsinki and was approved by the Institutional Review Board of Renmin Hospital of Wuhan University. Informed consent was obtained from all participants. The clinical research Ethics Review approval number of Renmin Hospital of Wuhan University was WDRY2022-K130.

### Statistical analysis

All statistical analyses were performed using SPSS 26.0 for Windows (SPSS, Inc.) The categorical variables are expressed as the number of cases (percentage), and the chi-squared test was used for comparisons between groups. The continuous variables are expressed as medians (range), and comparisons between the groups were performed using the Mann–Whitney U test (two groups) or the Kruskal–Wallis test (more than two groups). Cronbach’s α coefficient was used to test the internal consistency of the questionnaire. The Spearman-Brown coefficient was calculated by using the split-half reliability method to test the consistency of the questionnaire across items. The inter-time consistency of the questionnaire was tested using the retest reliability method, which was assessed by computing the intra-class correlation coefficient (ICC). A reliability coefficient of greater than 0.8 was considered excellent. Pearson’s correlation analysis was used to assess the correlation between indicators. A correlation coefficient (R) of greater than 0.7 was excellent. A *P* value of < 0.05 was considered statistically significant.

## Results

### Questionnaires revision and reliability and validity test results

A total of 15 experts were selected for consultation, 10 of whom were female, with an average age of 47.6 years (range 31–60). The average time working in the IBD field was 15 years (range 5–30), and they were from different cities in China. Fourteen specialists held a doctorate degree, and one expert held a master’s degree. Thirteen specialists had senior or senior affiliate titles. The response rate of the consultation questionnaire was 100%. Two items (B7 and C10) were removed according to the inclusion and exclusion criteria specified in the Methods. The results of the expert correspondence are shown in Supplementary Table 1.

Forty-one subjects participated in the pre-survey, among which, 17 (41.5%) were female, and 29 (70.7%) were CD patients with an average age of 31.7 (18–52) years. The effective recovery rate of the questionnaires was 100%, and the completion time was 15 to 20 min. The participants evaluated and gave feedback on the questionnaires. As a result, some difficult technical vocabulary terms were explained, imaging items were added, and the depth of individual items was improved based on patient feedback. For example, most CD patients knew that smoking was harmful, but they did not know the specific harm, so we modified the depth of the corresponding items. To avoid the upper limit effect, we removed items in the first version of the questionnaires in which the percentage of correct answers for B1 and B4 surpassed 95%. The final IBD knowledge questionnaires consisted of two versions: CD and UC. Each questionnaire contained 22 questions, 18 of which were shared by UC and CD. Three items reflecting the same topic in UC and CD knowledge questionnaires, including lesion site, biological indication, and surgical indication, were also processed as common items.

A total of 639 IBD patients were recruited to participate in the formal survey. After excluding 15 questionnaires from patients younger than 18, 624 valid questionnaires were collected. Two weeks later, 59 patients were invited to participate in the retest reliability analysis of the IBD knowledge questionnaires, which 44 patients completed.

The reliability analysis demonstrated that the reliability quality of the data was excellent. In the internal consistency test, Cronbach’s α coefficients for the common items, UC, and CD knowledge questionnaire were 0.886, 0.890, and 0.886, respectively. The internal consistency results of each dimension are shown in Table 1. In the split-half reliability test, the Spearman-Brown coefficient of the common items was 0.843, and the Spearman-Brown coefficient for the UC and CD knowledge questionnaire was both 0.812. In the inter-time consistency test, the ICC of the common items was 0.862 (*P* < 0.001).

**Table 1:** Internal consistency of IBD knowledge questionnaire.

Dimensionality	Common items(n = 624)		CD Knowledge Questionnaire (n = 429)		UC Knowledge Questionnaire (n = 195)	
	Cronbach’s alpha	number of terms	Cronbach’s alpha	number of terms	Cronbach’s alpha	number of terms
A	0.601	3	0.525	4	0.537	3
B	0.517	4	0.469	4	0.544	4
C	0.806	9	0.800	9	0.810	10
D	0.717	5	0.693	5	0.700	5
totality	0.886	21	0.872	22	0.890	22

A: pathology and risk factors, B: diet and nutrition, C: therapy, D: disease surveillance and special circumstances.

Regarding content validity, the contents of each item in the IBD knowledge questionnaires were obtained from a literature review. Then, the items were optimized through item sorting and analysis, expert qualitative review, and patient interviews to yield the IBD knowledge questionnaires, which should have excellent content validity. The patient’s assessment of the IBD knowledge questionnaires similarly displayed high agreement. The average score of “proper item difficulty” was 3.77 (CV was 0.19), the average score of “clear item expression” was 4.20 (CV was 0.15), and the average score of “comprehensive item content” was 3.89 (CV was 0.17), and the average score of “items help to understand IBD” was 4.02 (CV was 0.18). Regarding calibration correlation validity, the correlation coefficient (R) between the doctor score, IBD-KNOW score, and the first version of the questionnaires was 0.708 and 0.803 (*P* < 0.001; Supplementary Table 2), respectively, indicating a good correlation. The correlation between the scores on the initial questionnaires and patients’ self-scores was weak (r 0.556, *P* < 0.001; Supplementary Table 2), and there was a weak correlation between physician scores and the scores of all dimensions in the first version of the questionnaires (Supplementary Table 3).

### Current status of IBD patients’ understanding of disease and analysis of influencing factors

The mean (SD) age of the 624 IBD patients was 36.59 (12.49) years, of whom 234 (37.5%) were female. The general demographic characteristics of IBD patients are shown in Table 2.

**Table 2:** Demographic characteristics of patients with inflammatory bowel disease (N = 624).

Variables	N (%)	Variables	N (%)
Age(year)		Complication	
18–35	335 (53.7)	No	337 (54.0)
36–60	263 (42.2)	Yes	287 (46.0)
> 60	26 (4.17)	Influence on work/study	
Gender		Mild	335 (53.7)
Female	234 (37.5)	Moderate	191 (30.6)
Male	390 (62.5)	Severe	98 (15.7)
Character		Family history	
Introverted	362 (58.0)	No	582 (93.3)
Outgoing	163 (26.1)	Yes	42 (6.70)
Ordinary	99 (15.9)	Other chronic diseases	
Nation		No	443 (71.0)
Han	605 (97.0)	Yes	181 (29.0)
Minority	19 (3.04)	Aminosalicylic acid	
BMI		No	419 (67.2)
Underweight	129 (20.7)	Yes	205 (32.9)
Normal	338 (54.2)	Hormone	
Overweight	157 (24.8)	No	270 (43.3)
Education		Yes	354 (56.7)
Low	198 (31.7)	Immunosuppressor	
Intermediate	147 (23.6)	No	285 (45.7)
High	279 (44.7)	Yes	339 (54.3)
Residence		Biological agent	
City	458 (73.4)	No	464 (74.4)
Country	166 (26.6)	Yes	160 (25.6)
Solitary		EN	
Yes	62 (9.90)	No	441 (70.7)
No	562 (90.1)	Yes	183 (29.3)
Married		Surgery for IBD	
No	237 (38.0)	No	360 (57.7)
Yes	387 (62.0)	Yes	264 (42.3)
Employment		Duration	
Student	64 (10.3)	< 1 year	92 (14.7)
Yes	341 (54.6)	1–3 years	157 (25.2)
No	219 (35.1)	> 3 years	375 (60.1)
Medical insurance		Hospital visits (time/year)	
New rural cooperative medical insurance	107 (17.1)	< 3	170 (27.2)
Basic medical insurance for urban residents	468 (75.0)	3–5	147 (23.6)
Other	49 (7.9)	> 5	307 (49.2)
Income(RMB/month)		Hospital stay (week/year)	
< 5000	298 (48.7)	< 2	279 (44.7)
5000–10,000	197 (31.6)	2–4	148 (23.7)
≥ 10,000	129 (20.7)	> 14	84 (13.5)
Treatment expense(RMB/year)		Smoking status	
< 10,000	138 (22.1)	Yes	470 (75.3)
10,000–20,000	119 (19.1)	Have given up	112 (17.9)
≥ 20000RMB	367 (58.8)	No	42 (6.70)
Disease type		Drinking status	
UC	195 (31.3)	No	553 (88.6)
CD	429 (68.8)	Occasionally	53 (8.50)
Disease activity		Frequent	18 (2.9)
Remission	334 (53.5)	Exercise frequency	
Active	290 (46.5)	Few	203 (32.5)
Mild	180 (28.9)	Occasionally	209 (33.5)
Moderate	93 (14.9)	Frequently	212 (34.0)
Severe	17 (2.72)		

*BMI* body mass index.

**Figure 1:**
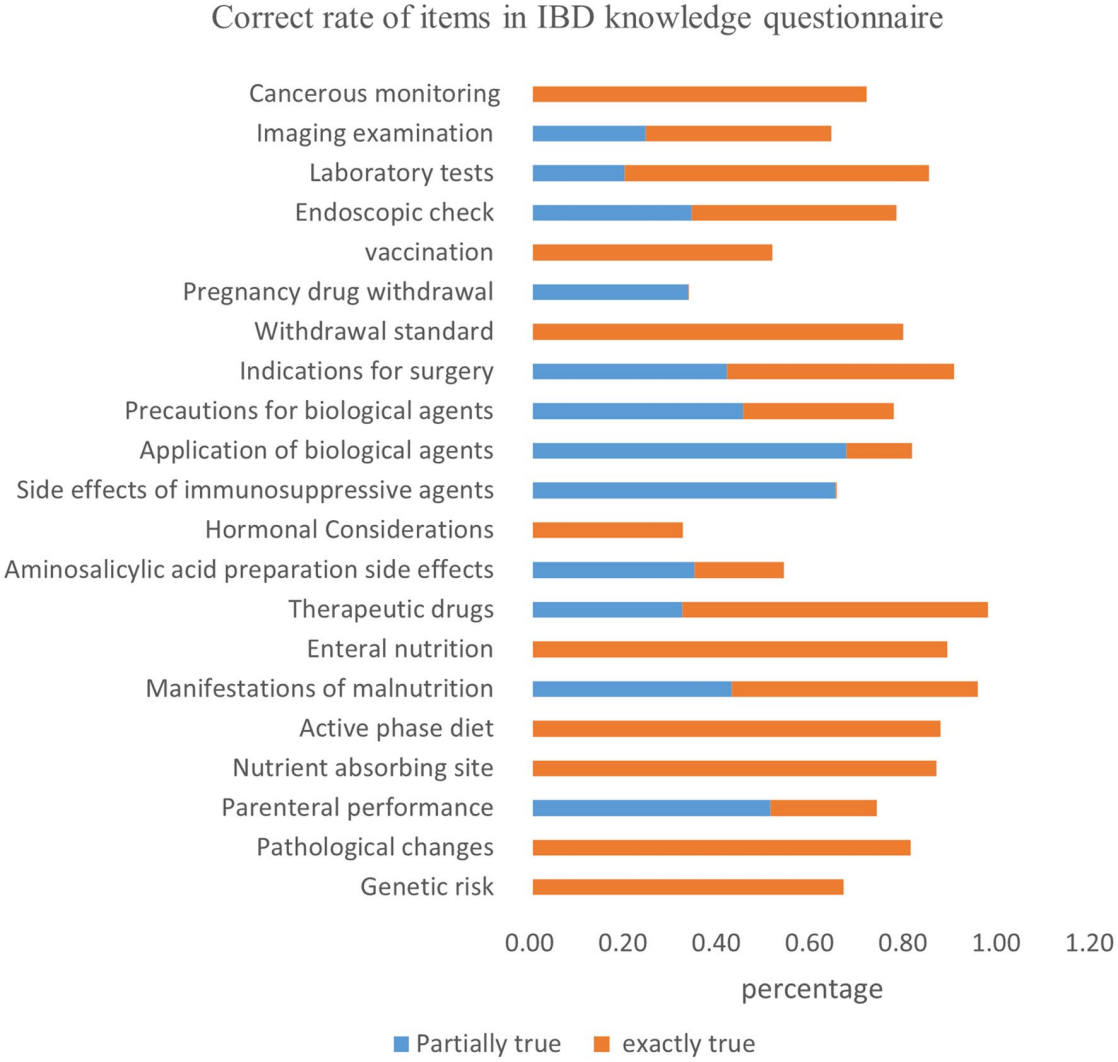
Correct response rate of patients to each item of the IBD knowledge questionnaire.

**Figure 2:**
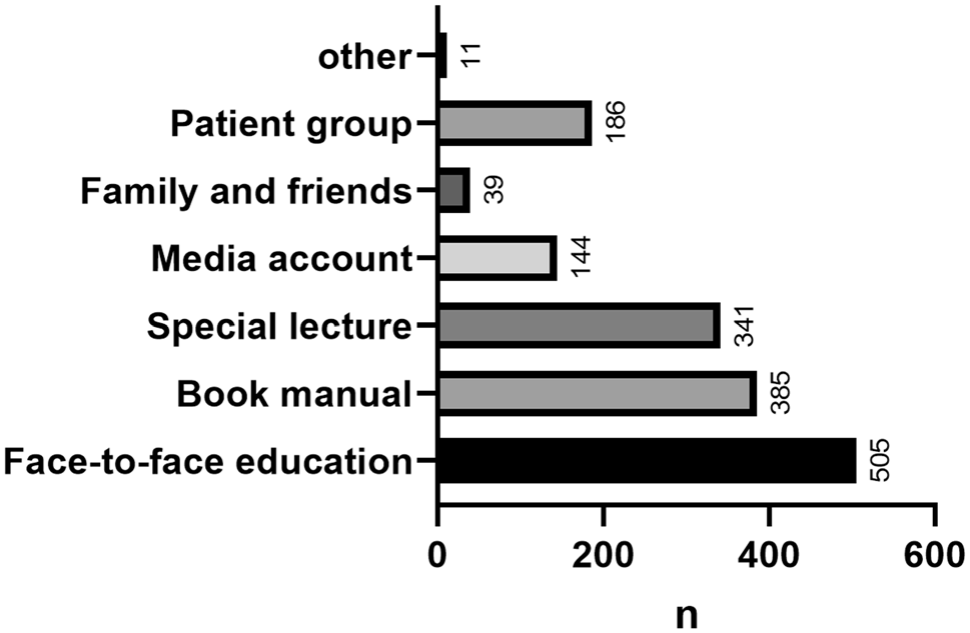
The main source of IBD information.

The knowledge of IBD patients about the disease is shown in Supplementary Table 4. The total scores of the UC knowledge questionnaire, CD knowledge questionnaire, and the common items of the IBD knowledge questionnaires were 34 points, 35 points, and 33 points, respectively. CD patients had better knowledge of IBD than UC patients (*P* < 0.001) (Supplementary Table 4). In the cognitive analysis of the common items, more than 50% of patients responded with incorrect answers to questions on topics related to hormone therapy and pregnancy treatment (Fig. 1). The main sources of disease knowledge for patients with IBD were face-to-face education, book or manuals, and special lecture (Fig. 2).

### Multiple linear regression analysis of factors influencing disease knowledge in IBD patients

Based on the single factor analysis (*P* < 0.05) (Supplementary Table 5), the indicators with statistically significant differences were used as independent variables, and the common items of the IBD knowledge questionnaires scores were used as dependent variables for multiple linear regression analysis. The results showed that disease type, age, BMI, education, income, treatment cost, duration of disease, and frequency of visits were independent factors influencing IBD patients’ disease knowledge (Table 3).

**Table 3:** The multivariate linear regression analysis of influencing factors of disease knowledge in patients with IBD.

Category	B	Beta	t	*p*	CI
Intercept	4.26		1.68	0.094	(− 0.73, 9.26)
Diagnosis					
UC	Reference				
CD	2.09	0.15	3.59	< 0.001	(0.95, 3.23)
Age	− 1.50	− 0.13	− 3.17	0.002	(− 2.43, − 0.57)
BMI	− 1.46	− 0.15	− 4.25	< 0.001	(− 2.13, − 0.79)
Education	2.24	0.29	7.09	< 0.001	(1.62, 2.86)
Married					
No	Reference				
Yes	0.34	0.03	0.58	0.561	(− 0.8, 1.47)
Residence					
City	Reference				
Rural	− 0.18	− 0.01	− 0.32	0.747	(− 1.27, 0.91)
Employment					
No	Reference				
Student	− 0.18	− 0.01	− 0.19	0.846	(− 1.94, 1.59)
Yes	0.32	0.02	0.59	0.556	(− 0.75, 1.4)
Income	0.68	0.08	2.03	0.043	(0.02, 1.33)
Treatment expense	1.06	0.13	3.68	< 0.001	(0.49, 1.62)
Disease activity					
Remission					
Activity	− 0.81	− 0.06	− 1.69	0.092	(− 1.74, 0.13)
Other chronic diseases					
No	Reference				
Yes	0.24	0.02	0.45	0.651	(− 0.79, 1.26)
Surgery for IBD					
No	Reference				
Yes	0.78	0.06	1.50	0.135	(− 0.24, 1.79)
Duration	1.53	0.17	4.85	< 0.001	(0.91, 2.15)
Hospital visits	0.64	0.08	2.34	0.019	(0.1, 1.18)

## Discussion

We developed culturally appropriate IBD knowledge questionnaires and conducted a multicenter prospective cross-sectional study (the formal survey questionnaire is attached as Supplementary Material and includes the UC and CD knowledge questionnaires). The content of the IBD knowledge questionnaires was evaluated and approved by 15 specialists in the field of IBD in China. The items on the questionnaires include pathology, risk factors, diet and nutrition, therapy, disease activity evaluation, and other scientific and comprehensive aspects.

Some items in the questionnaires were presented in the form of multiple choice questions, which could not only evaluate whether patients grasped the issues reflected in the items but also the extent of their understanding. This approach has unique advantages compared to previously published knowledge questionnaires for IBD patients. In addition, the questionnaires are comparatively brief and readily available, and the differences between CD and UC are considered. The internal consistency, split-half reliability, and test–retest reliability of the questionnaires were all good. In the effectiveness evaluation, the IBD knowledge questionnaires, the face-to-face evaluation of the study physicians, the correlation with the Chinese IBD‐KNOW^15^ questionnaire, and the patients’ agreement with the questionnaires showed good results. The IBD‐KNOW questionnaire is an updated IBD knowledge questionnaire based on CCKNOW, which has been verified in South Korea and the United States^20^. The correlation between the IBD knowledge questionnaires and patient self-assessment was slightly worse, which may be related to the subjectivity of patient self-assessment.

This study showed that young patients with higher education, longer disease course, and higher income scored higher on the IBD knowledge questionnaires, consistent with prior related studies^17,20,21^. It also demonstrated the discriminative ability of the IBD knowledge questionnaires. Patients with a high level of IBD knowledge questionnaire score also displayed a heavy disease burden, characterized by CD disease type, low BMI, high therapy cost, and high frequency of visits. Previous studies showed that compared to UC patients, CD patients have a more severe disease burden^22^, emaciation, and higher therapy costs. More frequent visits increase the disease burden and economic burden of patients, which further promotes the need for patients to understand IBD. Books or lectures were the main sources of IBD information. Previous studies in South Korea^15^ and Germany^23^ indicated that the main source of patients’ information about IBD was the Internet, which was different from our study. A study of Chinese Internet information found that the readability of existing network health information was at a low level^24,25^, and was hard for most target readers to read and grasp^26^. Thus, continuing to pay attention to the traditional model of doctor-patient education while enhancing the quality of online education will be our future effort. Unexpectedly, unlike previous reports^16,27,28^, disease activity and IBD-related surgical history were not associated with the degree of understanding of the disease in the IBD patients in our study. However, a significant association between medication adherence and disease knowledge was demonstrated in IBD patients in our study, consistent with previous studies^11,29^.

A correct understanding of IBD is the basis of patient self-management^30^. The evaluation of the patient’s current knowledge of the disease can be used for targeted health education to gain better educational effects^29,31^. In the separate analysis of each knowledge item, we observed that patients had poor knowledge of therapy during pregnancy. Given that a large proportion of IBD patients are young men and women of childbearing age, doctors should pay attention to this aspect of education during medical treatment. We also observed that patients had different degrees of insufficient knowledge about the indications and side effects of diverse therapy modalities, even among individuals with a history of utilizing them. Various types of IBD drugs are available, some of which have obvious adverse reactions ^32,33^. Therefore, it is necessary to provide adequate drug-related knowledge education and appropriate follow-up for patients to understand the treatment plan better and obtain better treatment effects.

IBD patients who participated in developing and validating the questionnaires were recruited from multiple Chinese IBD patient groups without any financial compensation, and all participation was voluntary. Although the overall response rate was comparatively poor, we still gained a significant sample size. Therefore, the results of this survey are credible and representative. Compared with previously published IBD patient knowledge questionnaires, some items were presented in the form of multiple choice questions, which could assess whether the patient understood the issues reflected in the items objectively, as well as the extent to which the patient understood them.

The limitations of this survey include that the questionnaire was conducted online, and patients who were not in the IBD patient group were not included, which may impact the representability of the findings. There was also a lack of comprehensive information on IBD, such as fewer items related to surgery, reproduction, and family planning, which may be important for specific patient groups.

## Conclusions

We developed and carefully validated IBD knowledge questionnaires, including two versions for CD and UC. The content had appropriate difficulty and good reliability and validity. We also translated the questionnaires into English, hoping that they could be a reference for other countries. In our survey, the disease knowledge of IBD patients was related to disease type, age, BMI, education, income, treatment cost, duration of disease, and frequency of visits.

## Supplementary Information

Supplementary Information 1.

Supplementary Information 2.

Supplementary Information 3.

Supplementary Information 4.

Supplementary Information 5.

Supplementary Information 6.

## Data Availability

The datasets used and/or analysed during the current study available from the corresponding author on reasonable request.
