# Supplementary material for: Development and validation of a questionnaire to test Chinese patients’ knowledge of inflammatory bowel disease
Source: Sci Rep. 2023 Apr 30;13:7061. doi: 10.1038/s41598-023-34286-6 (PMC10149500; doi:10.1038/s41598-023-34286-6)
Supplement: Supplementary file 1 — Supplementary Information 1. [file 41598_2023_34286_MOESM1_ESM.docx]

**Supplementary material**

**Research on the Cognition of Inflammatory Bowel Disease Patients**

**Part 1The general information**

1. Your gender:

○Male

○female

2. Your age: ___________

3. Ethnic

○ Han

○ minority, please specify _________________ *

4. Height _________cm, weight _________kg

5. Your personality

○ introverted

○ export-oriented

○ the general

6. Your residence:

○ city

○ the rural

7. Your living condition:

○ live alone

○ Living with spouse/parents/children

○ other, please specify _________________

8. Your highest degree:

○ Elementary School and below

○ Middle school

○ High school

○ College

○ Bachelor degree

○ Master degree or above

9. Your marital status

○ marry

○ married

○ divorce

○ Death of a spouse

10. Your current occupation:

○ students

○ Administrative and public institutions

○ Business Units

○ Industrial and commercial individuals/service industries

○ the farmer

○ unemployed

○ retired

○ other

11. Your medical insurance type is

○ New rural cooperative medical insurance

○ Basic medical insurance for urban workers

○ Basic medical insurance for urban residents

○ Medical insurance for college students

○ Commercial medical insurance

○ Public health insurance

○ No medical insurance

12. What is your diagnosis?

○ Crohn's Disease

○ Ulcerative colitis

○ Not classified

13. Your lesion site is (CD patient)

□ Upper digestive tract (esophagus, stomach)

□ Terminal ileum

□ back to the colon

□ colon

□ I don't know

13. Your lesion site is (UC patient)

□ the rectum

□ Left colon

□ the whole colon

□ I don't know

14. Does anyone in your family or friends have inflammatory bowel disease?

○ no

○ Yes, there are

15. The time of your diagnosis was ____ years _____ months; (You can fill in the approximate time)

16. In the past year, you have visited the hospital __________ times for inflammatory bowel disease

17. Do you currently have any other chronic medical conditions [multiple choice] *

□ high blood pressure

□ diabetes

□ fatty liver

□ depression

□ Chronic heart disease, including congenital heart disease

□ Chronic lung disease (excluding asthma)

□ Asthma -

□ Chronic kidney disease

□ Chronic liver diseases (excluding fatty liver)

□ Chronic neurological diseases

□ Chronic blood diseases

□ Rheumatic diseases

□ Malignant tumors

□ Other, please specify _________________*

□ No other chronic diseases

18. The impact of inflammatory bowel disease on your current work/study status

○ No impact -

○ Frequent absences due to inflammatory bowel disease

○ Unemployed or absent from school due to inflammatory bowel disease

○ has retired

○Long-term Unemployment (Cause of unemployment Unrelated to inflammatory bowel disease)

19. Your family's per capita monthly income is about (before tax)

○ Less than RMB 2,000

○ RMB 2,000 to 5,000 (Excluding 5,000)-

○ RMB 5,000 to 10,000 (Excluding 10,000)

○ RMB 10,000 to 20,000 (excluding 20,000)

○ RMB 20,000 or more

20. The amount of money you spent on treatment for inflammatory bowel disease in the past year (before reimbursement)

○ Less than RMB 2,000

○ RMB 2,000 to 5,000 (Excluding 5,000)-

○ RMB 5,000 to 10,000 (Excluding 10,000)

○ RMB 10,000 to 20,000 (excluding 20,000)

○ RMB 20,000 or more

21. Do you smoke?

○ Smoking, but not yet

○ I used to smoke, but I quit

○ Never smoked -

22. Do you drink?

○ Almost no drinking

○ Occasionally, but once a week at most

○ Drinking more than once a week

○ Drinking almost Every day

23. How often do you do physical exercise

○ No exercise

○ Exercise occasionally, but not more than once a week -

○ Exercise more than once a week, but not every day -

○ Exercise every day

24. The amount of time you usually have a physical exercise

○Less than half an hour -

○Half an hour to one hour (including one hour)

○One to two hours (including two hours) -

○ More than two hours

25. Your general health status is (UC patient)

[Enter numbers, 1= very poor，10=perfect，]*

1________________________________10

26. How many times did you use the toilet to defecate on an average day (24 hours) during the previous week?

○0-3 times/day

○4 to 6 times/day

○7-9 times/day

○ More than 9 times/day

27. During the previous week, on average, how many times per night did you get up to use the toilet to defecate? (UC patients)

○ never

○ 1-3

○ More than 3

28. In the previous week, have you had any urgent defecation, the degree of which is (UC patients)

○ No feeling of urgent defecation -

○ Defecation is urgent but controllable

○ Rapid defecation is difficult to control

○ Fecal incontinence

29. Have you had blood in the stool in the previous week, and the degree is (UC patient)

○ No blood in stool

○ Slightly dark or bloody stool

○ Sometimes you can see blood in your stool

○ Blood is often seen in the stool

25. Your general condition is (CD patient)

○ good

○ a bit poor

○ poor

○ Bad

○ very poor

26. Your current abdominal pain is :(CD patient)

○ no

○ mild

○ moderate

○ severe

27. Your present stools: loose stools________ times a day. If there is no loose stools or diarrhea, fill in 0. Crohn's disease, please (CD patient).

28. Do you currently have an abdominal mass (doctor's opinion) (CD patient)

□ no

□ there are suspicious

□ Confirm abdominal mass

□ Abdominal mass and tenderness

29. Do you currently have any of the following complications [multiple choice] *

□Arthritis

□ Iritis

□ Erythema nodosum

□ Pyoderma gangrenosum

□ Aphthar ulce

□ Anal fissure

□ Intestinal fistula, anal fistula

□ Abdominal abscess, perianal abscess

□ Other, please specify _________________*

□ None of the above

30. The following questions are to evaluate your treatment compliance. Please fill in [matrix scale questions] * according to the real situation

| Changed my diet as prescribed by the doctor | Always | usually | sometimes | never |
| --- | --- | --- | --- | --- |
| Follow medical advice to avoid bad living habits |  |  |  |  |
| Monitor my nutritional status |  |  |  |  |
| Follow the current treatment plan |  |  |  |  |
| Go to the hospital for regular review |  |  |  |  |

31. What are the main ways you learn about IBD? Please select them in order of how useful they are to your understanding of IBD (in order of multiple choices).

[] Health care workers face to face

[] Books, manuals, and other paper materials

[] Special lecture

[] Related websites, Weibo, and wechat public accounts,etc

[] Family, friends, or colleagues

[] Patients group

[] Other, please specify _________________

32. The medications you are using or have used [matrix scale questions] *

|  | In use | Used before | Not used |
| --- | --- | --- | --- |
| Mesalamine |  |  |  |
| Biological agents |  |  |  |
| Glucocorticoid(e.g. Methylprednisolone/prednisone/Medrol) |  |  |  |
| Immunosuppressants (e.g. Azathioprine, methotrexate, thalidomide, cyclosporine, etc.) |  |  |  |
| Enteral nutrition |  |  |  |
| Traditional Chinese medicine |  |  |  |

33. Have you had any surgery for inflammatory bowel disease [multiple choice] [Multiple choice]

○ I have had gastrointestinal surgery (including partial bowel resection, osteoplasty, etc.), several times _________________*

○ done crissum surgery (including anal fistula hanging line and crissum abscess incision drainage, etc.), made several _____________ *

○ None of these operations have been performed

**Part 2The IBD Knowledge Questionnaire**

1. Risk profile of children with IBD if their parents had IBD * [single choice] *

○ Sure to Suffer from inflammatory bowel disease

○The risk of inflammatory bowel disease increases, but the probability is low.

○I don't know *

2. The main lesion site of ulcerative colitis is * [single choice] *

○ large intestine

○ the small intestine

○ I don't know

2. Lesion site of Crohn's disease * [single choice] *

○ Includes any part of the digestive tract from the mouth to the anus

○ Small intestine, large intestine, and anus only

○ I don't know

3. In addition to the gut, what other sites can inflammatory bowel disease cause disease * [Multiple choice] *

□ Skin mucous membranes (e.g. oral mucosa)

□ joint

□ Eye -

□ courage

□ I don't know

4. The main site of absorption of nutrients in the intestine is * [single choice] *

○ the small intestine

○ the large intestine.

○ I don't know

5. During inflammatory bowel disease activity, patients should eat more foods rich in dietary fiber, such as whole grains, vegetables, and fruits [single choice] *

○ Yes

○ mistakes

○ I don't know

6. Common manifestations of malnutrition in patients with IBD include * [Multiple choice] *

□ Weight and muscle mass decline

□ Anemia -

□ Osteoporosis

□ I don't know

7. Enteral nutrition can not only improve the nutrition of patients with IBD, but also be beneficial for disease remission, and should be used as the first choice when IBD patients need nutritional support

○ Yes

○ mistakes

○ I don't know

8. Inflammatory bowel disease treatment drugs include * [multiple choice] *

□ Aminosalicylic acid preparations (including transverse amino amine pyridinium, Saratoazine, Balazine)

□ Hormones (including methylprednisolone, prednisone acetate, hydrocortisone, dexamethasone)

□ Immunosuppressive agents (including azathioprine, cyclosporine, 6-mercaptopurine, tacrolimus)

□ Biological agents (including: infliximab, adalimumab, ulsinumab, vedolizumab)

□ I don't know

9. Patients with the rectum or rectosigmoid lesions may be treated with suppositories or enemas * [Multiple choice] *

○ Yes

○ mistakes

○ I don't know

9. What are the risks of smoking for Crohn's disease? [Multiple choice] *

□ Reducing the effect of drug treatment

□ Increased surgical risk

□ Increased risk of postoperative recurrence

□ I don't know

10. The side effects of aminosalicylic acid preparations are * [Multiple choice] *

□ Nausea, vomiting, diarrhea, and other gastrointestinal reactions

□ a rash

□ headache

□ Renal impairment

□ I don't know

11. Hormones can be used for the treatment of active inflammatory bowel disease or for maintenance treatment in remission * [single choice] *

○ Yes

○ mistakes

○ I don't know

12. The side effects of immunosuppressants are * [Multiple choice] *

□ Cytopenia

□ Liver damage

□ I don't know

13. Biological agents are mainly used for * [multiple choice] *

□ Patients with mild symptoms

□ Patients who do not respond to hormone and immunosuppressive therapy

□ Patients with anal fistula, intestinal fistula, or prominent intestinal manifestations such as arthritis, pyoderma gangrenosum, erythema nodosum, etc

□ I don't know

14. The correct statements about the use of biological agents are * [Multiple choice] *

□ Screening for active infections, especially hepatitis B and tuberculosis, is required

□ Drug concentration and drug antibody monitoring are required

□ May increase the risk of tumor development

□ May increase the risk of infection

□ I don't know

15. Patients with ulcerative colitis should consider surgery for which of the following conditions * [Multiple choice] *

□ Medical treatment failed

□Toxic megacolon, intestinal perforation, gastrointestinal hemorrhage

□cancerous

□ I don't know

15. Consider surgery when presenting with Crohn's disease * [multiple choice] *

□ Medical treatment failed

□ Fibrous ileus

□ Complicated with intestinal perforation, intraperitoneal abscess, and massive gastrointestinal bleeding

□ cancerous

□ I don't know *

16. Patients with inflammatory bowel disease can stop treatment if they have been free of symptoms such as diarrhea, blood in the stool, or abdominal pain for several years * [single choice] *

○ Yes

○ mistakes

○ I don't know

17. Medications that patients with inflammatory bowel disease must discontinue when considering pregnancy are * [Multiple choice] *

□ Methotrexate

□ Thalidomide

□ All medicines are off limits

□ I don't know

18. The vaccines available to patients with inflammatory bowel disease when immunocompromised are * [single choice] *

○ Inactivated vaccine

○ Live attenuated vaccine

○ No vaccinations

○ I don't know

19. What is true about colonoscopy, colonoscopy, and capsule endoscopy is * [Multiple choice] *

□ Capsule endoscopy can see the lesions of the entire intestine. A mucosal biopsy cannot be performed.

□ colonoscopy can see the colon and a small portion of the small intestine at the end. A mucosal biopsy can be performed.

□ Colonoscopy combined with colonoscopy can see the lesions of the entire intestine. A mucosal biopsy can be performed.

□ I don't know

20. Laboratory tests that can assess activity in inflammatory bowel disease are * [multiple choice]

□ Blood C-reactive protein (CRP)

□ Calprotectin in feces

□ blood sedimentation

□ I don't know

21. Regarding magnetic resonance enterography (MRE) and CT enterography (CTE) scans, * [Multiple choice] * is the correct statement

□ Intestinal NMR has no radiation and takes a long time.

□ Intestinal CT is time-consuming and has radiation.

□ I don't know

22. Patients with inflammatory bowel disease for 8 to 10 years should be screened for colorectal cancer [single choice] *

○ Yes

○ mistakes

○ I don't know
