# Supplementary material for: Development and validation of a questionnaire to test Chinese patients’ knowledge of inflammatory bowel disease
Source: Sci Rep. 2023 Apr 30;13:7061. doi: 10.1038/s41598-023-34286-6 (PMC10149500; doi:10.1038/s41598-023-34286-6)
Supplement: Supplementary file 2 — Supplementary Information 2. [file 41598_2023_34286_MOESM2_ESM.doc]

**Supplementary Table 1**. Results of expert letter consultation

| Terms | Relevance | | | Importance Terms | | |
| --- | --- | --- | --- | --- | --- | --- |
|  | X¯ | S | CV | X¯ | S | CV |
| A1. Genetic risks | 4.13 | 0.81 | 0.20 | 4.13 | 0.88 | 0.22 |
| A2. Dangers of smoking in CD | 4.60 | 0.61 | 0.14 | 4.60 | 0.61 | 0.14 |
| A3. UC lesion site | 4.60 | 0.61 | 0.14 | 4.47 | 0.81 | 0.19 |
| A4. CD lesion site | 4.60 | 0.61 | 0.14 | 4.60 | 0.61 | 0.14 |
| A5. Extraintestinal manifestations | 4.60 | 0.61 | 0.14 | 4.53 | 0.72 | 0.16 |
| B1. Orthotrophy | 4.27 | 0.68 | 0.16 | 4.33 | 0.70 | 0.17 |
| B2. Nutrient absorption site | 4.07 | 0.85 | 0.22 | 4.00 | 0.89 | 0.23 |
| B3. Active phase diet | 4.40 | 0.61 | 0.14 | 4.33 | 0.60 | 0.14 |
| B4. Nutritional Assessment | 4.47 | 0.62 | 0.14 | 4.40 | 0.71 | 0.17 |
| B5. Manifestations of malnutrition | 4.27 | 0.77 | 0.19 | 4.33 | 0.70 | 0.17 |
| B6. Enteral nutrition | 4.53 | 0.72 | 0.16 | 4.60 | 0.61 | 0.14 |
| B7. Therapeutic effect of tonic herbs | 3.60* | 1.14 | 0.33* | 3.60* | 1.14 | 0.33* |
| C1. Types of treatment drugs | 4.67 | 0.60 | 0.13 | 4.60 | 0.61 | 0.14 |
| C2. Indications for enemas | 4.53 | 0.62 | 0.14 | 4.33 | 0.79 | 0.19 |
| C3. 5 - ASA side effects | 4.33 | 0.70 | 0.17 | 4.20 | 0.75 | 0.18 |
| C4. Precautions for hormone use | 4.27 | 0.85 | 0.21 | 4.33 | 0.87 | 0.21 |
| C5. Attention to immunosuppression | 4.40 | 0.71 | 0.17 | 4.40 | 0.71 | 0.17 |
| C6. Indications for biological agents | 4.13 | 0.81 | 0.20 | 4.13 | 0.88 | 0.22 |
| C7. Precautions for biological agents | 4.40 | 0.61 | 0.14 | 4.40 | 0.61 | 0.14 |
| C8. Indications for UC surgery | 4.47 | 0.72 | 0.17 | 4.40 | 0.80 | 0.19 |
| C9. Indications for CD surgery | 4.47 | 0.72 | 0.17 | 4.40 | 0.80 | 0.19 |
| C10. The importance of a good attitude | 3.87* | 0.88 | 0.24 | 3.93* | 0.85 | 0.22 |
| C11. Withdrawal standard | 4.40 | 0.61 | 0.14 | 4.40 | 0.61 | 0.14 |
| C12. Tummy Honey | 4.47 | 0.72 | 0.17 | 4.47 | 0.72 | 0.17 |
| D1. enteroscopy | 4.13 | 0.88 | 0.22 | 4.13 | 0.96 | 0.24 |
| D2.Laboratory test index | 4.40 | 0.71 | 0.17 | 4.40 | 0.71 | 0.17 |
| D3.vaccination | 4.20 | 0.75 | 0.18 | 4.13 | 0.81 | 0.20 |
| D4.Cancerous monitoring | 4.33 | 0.70 | 0.17 | 4.33 | 0.70 | 0.17 |

X¯: Mean, S: standard deviation, CV: coefficient of variation; A: pathology and risk factors, B: diet and nutrition, C: therapy, D: disease surveillance and special circumstances; * Entries were excluded if their relevance or importance was less than 4, or if their coefficient of variation was greater than or equal to 2.5.
