# Supplementary material for: Development and validation of a questionnaire to test Chinese patients’ knowledge of inflammatory bowel disease
Source: Sci Rep. 2023 Apr 30;13:7061. doi: 10.1038/s41598-023-34286-6 (PMC10149500; doi:10.1038/s41598-023-34286-6)
Supplement: Supplementary file 3 — Supplementary Information 3. [file 41598_2023_34286_MOESM3_ESM.docx]

**Supplementary Table 2**.The correlation among Pearson Preliminary questionnaire scores, physician assessment scores, IBD-KNOW scores, and patient self-scores.

|  | Score the first version of the questionnaire | The doctor score | IBD-KNOW score | self score |
| --- | --- | --- | --- | --- |
| Score the first version of the questionnaire | 1 |  |  |  |
| The doctor score | 0.708** | 1 |  |  |
| IBD-KNOW score | 0.803** | 0.592** | 1 |  |
| self score | 0.556** | 0.747** | 0.612** | 1 |

** Significant correlation at 0.01 level (two-tailed) (P<0.0010).
