# Supplementary material for: Development and validation of a questionnaire to test Chinese patients’ knowledge of inflammatory bowel disease
Source: Sci Rep. 2023 Apr 30;13:7061. doi: 10.1038/s41598-023-34286-6 (PMC10149500; doi:10.1038/s41598-023-34286-6)
Supplement: Supplementary file 4 — Supplementary Information 4. [file 41598_2023_34286_MOESM4_ESM.docx]

**Supplementary Table 3**. The correlation between the dimensions of the initial questionnaire scores and the physician scores.

| Dimension | r | *P* value |
| --- | --- | --- |
| A pathology and risk factors | 0.609 | <0.001 |
| B diet and nutrition | 0.308 | <0.05 |
| C therapy | 0.637 | <0.001 |
| D disease surveillance and special circumstances | 0.568 | <0.001 |
