# Supplementary material for: Development and validation of a questionnaire to test Chinese patients’ knowledge of inflammatory bowel disease
Source: Sci Rep. 2023 Apr 30;13:7061. doi: 10.1038/s41598-023-34286-6 (PMC10149500; doi:10.1038/s41598-023-34286-6)
Supplement: Supplementary file 5 — Supplementary Information 5. [file 41598_2023_34286_MOESM5_ESM.docx]

**Supplementary Table 4.** The scores of each dimension of the IBD knowledge questionnaire.

| Dimensionality | Score ranges |  | M(range) | | Statistic *Z* | *P* value |
| --- | --- | --- | --- | --- | --- | --- |
|  |  | totality | UC | CD |  |  |
| A | 0-4 | 3(0-4) | 2(0-4) | 3(0-4) | 4.32 | <0.001 |
| B | 0-5 | 4(0-5) | 4(0-5) | 4(0-5) | 4.37 | <0.001 |
| C | 0-16 | 8(0-14) | 7(0-13) | 8(0-14) | 4.43 | <0.001 |
| D | 0-8 | 5(0-8) | 4(0-8) | 6(0-8) | 8.29 | <0.001 |

A:pathology and risk factors, B: diet and nutrition, C: therapy, D: disease surveillance and special circumstances; M: Median
