# Supplementary material for: Development and validation of a questionnaire to test Chinese patients’ knowledge of inflammatory bowel disease
Source: Sci Rep. 2023 Apr 30;13:7061. doi: 10.1038/s41598-023-34286-6 (PMC10149500; doi:10.1038/s41598-023-34286-6)
Supplement: Supplementary file 6 — Supplementary Information 6. [file 41598_2023_34286_MOESM6_ESM.docx]

**Supplementary Table 5** Single factor analysis of influencing factors of disease knowledge in patients with IBD

| **Variables** | **Median (range)** | ***p*** | **Variables** | **Median (range)** | ***p*** |
| --- | --- | --- | --- | --- | --- |
| **Age(year)** |  | <0.001 | **Treatment expense(RMB/year)** | | <0.001 |
| 18-35 | 22(4-31) |  | <10000 | 17(2-29) |  |
| 36-60 | 19(0-30) |  | 10000-20000 | 18(1-30) |  |
| >60 | 14.5(2-28) |  | ≥20000 | 22(0-31) |  |
| **Gender** |  | 0.86 | **Disease type** |  | <0.001 |
| Female | 21(1-31) |  | UC | 17(1-30) |  |
| Male | 20(0-31) |  | CD | 22(0-31) |  |
| **Character** |  | 0.077 | **Disease activity** |  | 0.002 |
| Introverted | 20(0-30) |  | Remission | 22(1-31) |  |
| Outgoing | 22(1-31) |  | Active | 19(0-30) |  |
| Ordinary | 19(1-31) |  | **Complication** | | 0.062 |
| **Nation** |  | 0.205 | No | 20(0-31) |  |
| Han | 21(0-31) |  | Yes | 21(2-30) |  |
| Minority | 18(4-27) |  | **Influence on work/study** | | 0.099 |
| **BMI** |  | <0.001 | Mild | 20(1-31) |  |
| Underweight | 22(5-30) |  | Moderate | 21(1-31) |  |
| Normal | 21(1-31) |  | Severe | 20(0-30) |  |
| Overweight | 18(0-31) |  | **Family history** |  | 0.166 |
| **Education** |  | <0.001 | No | 21(0-31) |  |
| Low | 16(0-30) |  | Yes | 18(5-29) |  |
| Intermediate | 20(1-30) |  | **Other chronic disease** | | 0.035 |
| High | 23(4-31) |  | No | 21(0-31) |  |
| **Residence** |  | 0.004 | Yes | 20(1-30) |  |
| City | 21(1-31) |  | **Surgery for IBD** |  | <0.001 |
| Country | 18(0-29) |  | No | 19(1-31) |  |
| **Solitary** |  | 0.187 | Yes | 22(0-31) |  |
| Yes | 21.5(1-30) |  | **Duration** |  | 0.001 |
| No | 20(0-31) |  | < 1 year | 18(1-30) |  |
| **Married** |  | 0.002 | 1-3years | 19(0-31) |  |
| No | 21(1-31) |  | > 3years | 21(2-31) |  |
| Yes | 20(0-31) |  | **Hospital visits(time/year)** | | <0.001 |
| **Employment** |  | <0.001 | <3 | 18(2-30) |  |
| Student | 21(7-29) |  | 3-5 | 19(1-30) |  |
| Yes | 21(1-31) |  | > 5 | 22(0-31) |  |
| No | 18(0-30) |  | **Hospital stay(week/year)** | | 0.158 |
| **Income(RMB/month)** | | <0.001 | < 2 | 21(1-31) |  |
| <5000 | 18(0-30) |  | 2 -4 | 19(0-31) |  |
| 5000-10000 | 21(2-30) |  | > 4 | 20(3-30) |  |
| ≥10000 | 23(1-31) |  |  | |  |

BMI：Body mass index
